# Draft Genome Sequence of a Novel *Coriobacteriaceae* sp. Strain, EMTCatB1, Reconstructed from the Metagenome of a Thermophilic Electromethanogenic Biocathode

**DOI:** 10.1128/genomeA.00022-17

**Published:** 2017-03-09

**Authors:** Hajime Kobayashi, Qian Fu, Haruo Maeda, Kozo Sato

**Affiliations:** a Department of System Innovation, Graduate School of Engineering, University of Tokyo, Tokyo, Japan; b Engineering for Sustainable Carbon Cycle (INPEX Corporation) Social Cooperation Program, Frontier Research Center for Energy and Resources (FRCER), University of Tokyo, Tokyo, Japan; c INPEX Corporation, Tokyo, Japan

## Abstract

A draft genome of *Coriobacteriaceae* sp. strain EMTCatB1 was determined through taxonomic binning of a metagenome of a thermophilic biocathode actively catalyzing electromethanogenesis. This genome will provide information about the biocathode ecosystem, as well as the natural diversity of the *Coriobacteriaceae* family.

## GENOME ANNOUNCEMENT

Electromethanogenesis is a bioelectrochemical process, in which direct reduction of CO_2_ to methane is catalyzed by biocathodes (1). Recent studies have indicated that methanogens play a central role in the biocathode. However, the function(s) of other microbes colonizing on the biocathode have never been examined. The draft genome reported here was obtained from shotgun sequencing of the metagenome of thermophilic biocathodes actively catalyzing electromethanogenesis at 55°C for more than 60 days at a poised potential of −0.5 V versus the standard hydrogen electrode (2).

Sequencing of the metagenome was performed using an Illumina HiSeq 2000 platform (150-bp paired-end sequencing, two lanes). Sequence reads were trimmed after removal of adapter sequences by Cutadapt version 1.8.3 (3). Approximately 395 million trimmed reads (approx. 60 Gb) were used for phylogenetic and functional profiling of the metagenome. An initial metagenomic binning indicated that approximately 60% of the read sequences were derived from a single operational taxonomic unit, suggesting that the corresponding organism (named “EMTCatB1”) plays a role in the biocathode ecosystem. To assemble the sequences from the microbe, the reads were down-sampled to 400 Mb, thereby reducing sequences originating from relatively minor species, and assembled using Velvet, followed by gap-closing with Sealer (4, 5). The resulting draft genome of strain EMTCatB1 is 1.84 Mb (G+C content, 67.2%) contained in a single circular scaffold with no gap, representing a circular chromosome. The scaffold was annotated with the Genaris Annotation System (Genaris, Inc., Kanagawa, Japan) (3), revealing a total of 1,710 features (1,660 protein-coding genes and 50 RNAs). Among the 1,660 proteins, 1,310 (79%) were assigned to different functional categories of NCBI Clusters of Orthologous Groups (COG) (6). The most abundant COG category was “Amino acid metabolism and transport” (132 proteins), followed by “General functional prediction only” (126 proteins) and “Translation” (126 proteins).

Phylogenetic analysis of the 16S and 23S rRNA genes and RpoB revealed that strain EMTCatB1 represents a distinct lineage deeply rooted within the family* Coriobacteriaceae* of the phylum* Actinobacteria*, showing, respectively, 88 to 90%, 82 to 85%, and 70 to 73% sequence identities to the cultured representatives, and thus likely belongs to a new genus. The members of *Coriobacteriaceae* are obligate anaerobes capable of fermentation or anaerobic respiration (7). The latter metabolism is likely retained in the genome, which encodes homologs of enzymes required for hydrogen and formate oxidation (hydrogenases and formate dehydrogenases). Interestingly, those enzymes can mediate direct electron uptake at the cathode (8). Additionally, the draft genome encodes many putative redox proteins (18 c-type cytochromes and 24 ferredoxins) possibly involved in the electron transfer (9).

Members of the *Coriobacteriaceae* family are dominant bacteria of human gut microbiota and gain an increasing attention due to their roles in host metabolisms and as pathobionts (10). This is the first genome of *Coriobacteriaceae* from a non-host-related habitat and will be useful for understanding the biocathode ecosystem and diversity of the *Coriobacteriaceae* family.

### Accession number(s).

The *Coriobacteriaceae* sp. strain EMTCatB1 draft genome reported here is available in the DDBJ/EMBL/GenBank database under the accession number BDLO01000001.

## References

[1] Cheng S, Xing D, Call DF, Logan BE 2009 Direct biological conversion of electrical current into methane by electromethanogenesis. Environ Sci Technol 43:3953–3958. doi:10.1021/es803531g. 19544913

[2] Fu Q, Kuramochi Y, Fukushima N, Maeda H, Sato K, Kobayashi H 2015 Bioelectrochemical analyses of the development of a thermophilic biocathode catalyzing electromethanogenesis. Environ Sci Technol 49:1225–1232. doi:10.1021/es5052233. 25544349

[3] Nishi T, Ikemura T, Kanaya S 2005 GeneLook: a novel ab initio gene identification system suitable for automated annotation of prokaryotic sequences. Gene 346:115–125. doi:10.1016/j.gene.2004.10.018. 15716020

[4] Paulino D, Warren RL, Vandervalk BP, Raymond A, Jackman SD, Birol I 2015 Sealer: a scalable gap-closing application for finishing draft genomes. BMC Bioinformatics 16:230. doi:10.1186/s12859-015-0663-4. 26209068 PMC4515008

[5] Zerbino DR, Birney E 2008 Velvet: algorithms for de novo short read assembly using de Bruijn graphs. Genome Res 18:821–829. doi:10.1101/gr.074492.107. 18349386 PMC2336801

[6] Tatusov RL, Galperin MY, Natale DA, Koonin EV 2000 The COG database: a tool for genome-scale analysis of protein functions and evolution. Nucleic Acids Res 28:33–36. doi:10.1093/nar/28.1.33. 10592175 PMC102395

[7] König H 2015 *Coriobacteriia* class. nov., p. 1975–2001. *In* Bergey’s manual of systematics of archaea and bacteria. John Wiley & Sons, Ltd, New York. doi:10.1002/9781118960608.cbm00005.

[8] Deutzmann JS, Sahin M, Spormann AM 2015 Extracellular enzymes facilitate electron uptake in biocorrosion and bioelectrosynthesis. mBio 6:e00496-15. doi:10.1128/mBio.00496-15. 25900658 PMC4453541

[9] Choi O, Sang BI 2016 Extracellular electron transfer from cathode to microbes: application for biofuel production. Biotechnol Biofuels 9:11. doi:10.1186/s13068-016-0426-0. 26788124 PMC4717640

[10] Clavel T, Desmarchelier C, Haller D, Gérard P, Rohn S, Lepage P, Daniel H 2014 Intestinal microbiota in metabolic diseases: from bacterial community structure and functions to species of pathophysiological relevance. Gut Microbes 5:544–551. doi:10.4161/gmic.29331. 25003516

